# Prevalence and Related Factors of Post-Traumatic Stress Disorder in Emergency Medical Technicians; a Cross-sectional Study

**DOI:** 10.22037/aaem.v9i1.1157

**Published:** 2021-04-30

**Authors:** Afshin Khazaei, Elham Navab, Maryam Esmaeili, Habib Masoumi

**Affiliations:** 1 Intensive Care and Management Nursing Department, School of Nursing and Midwifery, Tehran University of Medical Sciences, Tehran, Iran.; 2 Critical Care Department, Nursing and Midwifery Care Research Center, School of Nursing and Midwifery, Tehran University of Medical Sciences, Tehran, Iran.; 3 Disaster and Emergency Management Department, Hamadan University of Medical Sciences, Hamadan, Iran.; 4 Critical Care and Geriatric Nursing Department, School of Nursing and Midwifery, Tehran University of Medical Sciences, Tehran, Iran.

**Keywords:** Emergency medical technicians, emergency medical services, diagnostic and statistical manual of mental disorders, stress disorders, post-traumatic

## Abstract

**Introduction:**

Ongoing exposure to a variety of Pre-hospital Emergencies (PE) has placed Emergency Medical Technicians (EMTs) at serious psychiatric compromise such as Post-Traumatic Stress Disorder (PTSD). The present study aimed to evaluate the prevalence and associated factors of PTSD among EMTs.

**Methods:**

This prospective cross-sectional study was conducted on EMTs in the Emergency Medical Services (EMS) in west of Iran. A baseline information questionnaire including personal work-related characteristics and the PTSD checklist of DSM-5 (PCL-5) were used for data collection. Non-parametric tests and multivariate linear regression were used to evaluate the associated factors of PTSD in these participants.

**Results:**

Among the participants, 22% of technicians had PTSD-diagnostic criteria. The mean total PCL-5 score was 21.60 ± 11.45, while the scores were 38.02 ± 6.08 and 17.47 ± 8.36 in the PTSD-diagnosed and undiagnosed groups, respectively. The most common symptom of the clusters was negative alterations in cognition with a mean score of 7.42 ± 4.63. After adjusting confounders, the number of missions (t= 2.50, P= 0.013), work experience (t= -3.24, P= 0.001) and number of shifts (t: 26.38, P < 0.001) were significantly corelated with PCL-5 score.

**Conclusion:**

The results indicated that the prevalence of PTSD among EMTs personnel of Hamedan province is high. EMTs with the age of ≤ 30 years, work experience of ≤ 10 years, married status, informal employment, emergency medical technician's degree, and more than 8 shifts per month, as well as no previous training history had a higher total PCL-5 score.

## Introduction

Emergency Medical Technicians (EMTs) experience some cumulative stress, which may be related to traumatic events (1). In addition, frequent and ongoing exposure to potentially traumatic events may place EMTs at higher risk of serious psychiatric compromise, including Post-Traumatic Stress Disorder (PTSD) (2, 3), which is considered as a mental health disorder leading to social, occupational, and interpersonal disturbance (4). 

The experience of a traumatic event is not the only effective factor causing PTSD among individuals (5). Therefore, identifying risk factors other than exposure to traumatic events, such as personal/work-related characteristics that can predict the development of PTSD, can lead to more effective management and control of prehospital emergency stress in the EMTs.

Considering the current overall PTSD prevalence (from 11% to 35%) among EMTs (6, 7), which is the highest rate among prehospital care providers (8), the need for assessing the mental health of EMTs and identifying the staff members at high risk of developing PTSD is crucial.  

Although a large number of studies have examined PTSD in EMTs using DSM-4 tools, little information is available on assessing PTSD by considering DSM-5 criteria in the EMS. Therefore, the present study aimed to evaluate the prevalence and associated factors of PTSD among EMTs.

## Methods

***Study design and setting***

In this cross-sectional study, the data of EMTs in 20 metropolitan-based, 30 road-based, and one air-based services (serving about two million people) in Hamadan province, Iran, were collected during July-October 2018. All of the EMTs in the Emergency Medical Services (EMS) in this province were invited to participate in this study. Before running the study, the objective of the study was explained to the EMTs. Then, all participants voluntarily signed the consent form and their names and personal information were kept confidential in the questionnaires. The project was approved by the Ethics Committee in Tehran University of Medical Sciences (No: IR.TUMS.FNM.REC.1397.042). 

***Participants***

Operational EMTs who worked in urban, road, and air emergency bases full-time and gave their oral and written consent were included in the presnet study. However, non-operational EMTs, the staff from other medical centers working part-time, and those who experienced non-occupational stressors such as the death of close relatives in the previous eight weeks were excluded.

***Data Gathering***

In order to collect the related data, demographic questionnaire (including personal and work-related characteristics) and PTSD Checklist for Diagnostic and Statistical Manual of Mental Disorders-5 (PCL-5), which is regarded as a self-reporting tool that evaluates a variety of purposes such as screening individual for PTSD, were used in this study (10). The PCL5 checklist includes 20 items, divided into four clusters including intrusion (5 items), avoidance (2 items), negative alterations in cognition and mood (7 items) and alterations in arousal and reactivity (7 items). Each item is scored on a 5-point Likert scale ranging from 0 (Not at all) to 4 (Extremely) (10). Furthermore, the reliability and validity of this checklist have been confirmed in some studies (11-13). Although the validity and reliability of the Persian version of this instrument were confirmed through using exploratory and confirmatory factor analyses, convergence validity (r=0.68%, P=0.001), and Cronbach’s alpha (r=0.79%), as well as retesting (r=0.77%) (14), we reassessed the PCL-5 reliability (r=0.89) for the total score in this study.

**Statistical analysis**

In the study conducted by Iranmanesh et al. (9), the reported PTSD rate among EMTs was 0.22%. The total number of EMTs was 307, among whom 251 were selected by considering the relative error of 10% and 95% confidence interval. Continuous variables were expressed as mean and standard deviation (SD) or median and interquartile ranges (IQRs). Categorical variables were reported in frequency and percentages. The total score of symptom severity was obtained by summing the scores related to the 20 items, and ranged from 0 to 80. In addition, a PCL-5 score of less than 33 appears to not require further psychometric work (10, 15, 16). Therefore, scores were dichotomized into scores ≥ 33 (meeting the criteria for PTSD) and scores < 33 (not meeting the criteria for PTSD) for screening PTSD symptoms.

Kruskal-Wallis and Mann-Whitney tests, as well as multivariate linear regression (using OLS), were used for assessing the correlation and identifying the predictors for PTSD symptoms. Furthermore, interaction and multi-collinearity (Variance Inflation Factor < 10 or Torrance > 0.2) were assessed for the regression final model. Adjusted beta coefficients were computed based on 95% confidence intervals. Furthermore, model fits were evaluated using Scatterplots, Homoscedasticity, Durbin-Watson test, Normal P-P Plot, Q-Q plot, and Cook’s Distance values. Continuous variables such as age, work experience, number of shifts, and number of missions, and categorical variables such as marital status (single, married, divorced), degree (emergency medical technician, nurse, operation room technician, anesthesia technician), employment status (formal, informal), base location (urban, road, air) were considered as possible independent variables of the model. All statistical analyses were performed using IBM SPSS Statistics version 20 and P < 0.05 was considered as the significance level. 

## Results

**Figure 1:**
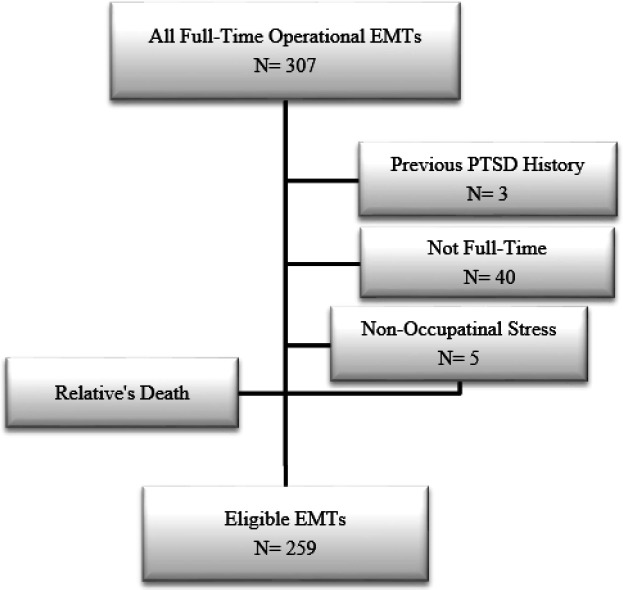
Study flowchart. EMTs: Emergency Medical Technicians

In the present study, 259 male EMTs were recruited for participation in the study after being qualified for the inclusion criteria (figure 1: study flowchart). The mean age of the participants was 32.79 ± 6.16 years (21 - 52) and their median work experience was 9 years (IQR 5-12). The median number of work shifts and pre-hospital missions in which technicians were deployed in the previous month was 12 (IQR 11-13) and 60 (IQR 9-85), respectively. 53.7% of the EMTs had previous training on stress control and management. 

PTSD prevalence in the EMTs was 22.00%. The mean age of EMTs in the PTSD-diagnosed group was 28.88 years (SD= 6.94) with mean total PCL-5 score of 38.02 (SD= 6.08), while mean age was 33.77 years (SD= 5.55) and mean total PCL-5 score was 17.47 (SD = 8.36) in the group without PTSD. The mean total PCL-5 score in all samples was 21.60 (SD = 11.45) and ranged from 4 to 50. In addition, the mean total score was 4.98 (SD= 3.08), 2.25 (SD= 1.70), 7.42 (SD= 4.63), and 6.94 (SD= 4.10) for intrusion, avoidance, negative alterations in cognition, and alterations in arousal and reactivity clusters, respectively (table 1).

Table 2 indicates the mean of each cluster and the total PCL-5 score based on personal/work-related characteristics.

Furthermore, negative alterations in cognition were regarded as the most common cluster symptom and ranged between a score of 2 (19.7%) to 22 (0.4%) based on intensity (6 items with a score between 0-24). As shown in Table 1, alterations in arousal and reactivity were the second most common symptoms with the score ranging between 2 (18.1%) and 19 (4.0%) (7 items with a score between 0-28). Furthermore, the result of Mann-Whitney test indicated that the difference between mean score of clusters in PTSD and non-PTSD groups was statistically significant (p<0.001).

Based on the results, demographic factors such as age (t =41.86, df=2, P<0.001), marital status (t =49.60, df=2, P<0.001) and number of shifts (Z= -6.78, P< 0.001) were significantly associated with PCL-5 score (Table 2). However, no significant relationship was observed between some factors such as work experience (t =3.01, df=2, P=0.204), number of mission (Z= -1.65, P= 0.098), employment status (Z= -1.07, p=0.282), base location (t =3.84, df=2, P=0.146), degree (t =0.42, df=3, P=0.935), and previous training history status (Z= -0.88, p=0.375) with the total PCL-5 score. 

After adjusting confounders in multivariate linear regression, the number of missions (t: 2.50, P=0.013) and work experience (t: -3.24, P=0.001) could significantly predict PTSD status. In this regard, the number of shifts per month was the strongest factor (t: 26.38, P<0.001). Additionally, no violation of assumptions was observed in the regression model. Finally, the linear regression of the final model was significant (f= 297.30, df= 258, P < 0.001), which could explain 77.8% of the variance in the total PCL-5 score (R2 = 0.77).

## Discussion

The present study aimed to screen those with PTSD according to DSM-5 criteria and identify its related factors among the studied EMTs. PTSD prevalence rate among the studied EMTs was 22.00%. In addition, negative alterations in cognition (M= 7.42) and avoidance (M= 2.25) were the most and least common clusters, respectively. In general, EMTs with the age of ≤ 30 years, work experience of ≤ 10 years, married status, informal employment, emergency medical technician's degree, and more than 8 shifts per month, as well as no previous training history had a higher total PCL-5 score.

Furthermore, the rate of PTSD among the EMTs in the present study (DSM-5) is considerably high, compared to that of other studies (22). Petrie et al., in their systematic review and meta-analysis, showed that the prevalence rate of PTSD (DSM-4) among ambulance staff was 11% (22). Fjeldheim et al. reported that 94% of paramedic trainees in a South African University were directly exposed to trauma, and only 16% met the diagnostic criteria for PTSD (23). Perhaps, the choice of different instruments and the context are some of the reasons for this variation in the reported prevalence of PTSD. 

**Figure 2:**
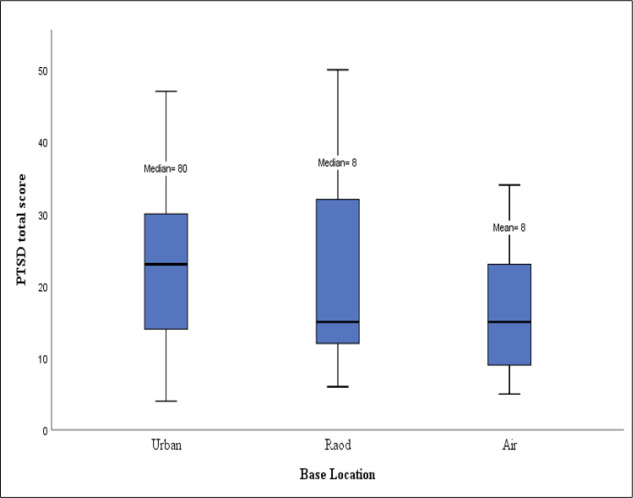
Simple Box Plot of correlation between base location and total PCL-5 score; Median: number of missions

The number of missions conducted by technicians stationed at the road and air bases was less than that of the urban bases, leading to less exposure to prehospital emergencies; yet, no significant difference was observed in the total PCL-5 score among the EMTs stationed at the three above-mentioned bases (P= 0.146) (figure 2). Perhaps, technicians who were stationed at the road and air bases have had more exposure to extremely traumatic events and provided high-acuity care in the unstable physical and environmental situations related to these bases such as the long distance between the road base with the first medical center, air turbulences, flight altitude, and the like. Presently, EMS transports those with a life-threatening condition and requiring critical care to a hospital via air bases. They experience more stress because the roads in Iran are considered to have one of the highest rates of accidents in the world and road-based technicians are faced with dangerous accidents leading to more casualties and injuries. The results of the present study were inconsistent with those of Schiszler et al., which reported that ground rescue workers are exposed to higher work-related stress compared to the air-ambulance workers (24).

Based on univariate linear regression, the number of shifts per month has a strong effect on the PCL-5 score so that the total PCL-5 score in the EMTs increased nearly 0.8% (standardized beta coefficient) in the exchange for doing a shift (24 h). The result is inconsistent with the study of Iranmanesh, which indicated that paramedics who work less than 100 hours per month may have a higher rate of PTSD (P=0.001) compared to those working 100–150 or more than 200 hours per month (9). In this regard, Shift Work Sleep Disorder (SWSD) may be regarded as one of the reasons that can explain the impact of shift work on individuals' PTSD. SWSD is considered a condition resulting from working atypical shifts including nights and long work hours, such as EMTs’ shifts, leading to the disorder of circadian rhythm and accordingly PTSD symptoms (25, 26).

 Furthermore, age was considered as another factor, which was significantly correlated with the PTSD score as a categorical and continuous variable in the Kruskal-Wallis (P<0.001) and the univariate linear regression (β =-0.36, t= -3.19, P=0.02) test. However, it was not regarded as a strong independent predictor of PTSD in the multivariate linear regression analysis. Kerai et al. found a negative relationship between age and PTSD symptoms (β = −0.17, P= 0.03) in the linear regression, which indicates a higher prevalence of PTSD in the younger staff (18). In the present study, the PTSD total score in technicians who were less than 30 years was higher than the score in other age groups (Table 2). Unexpectedly, the total score in the age group of 40 years was higher than that of 31-40 years in the EMT. Thus, age can be a protective factor against PTSD to a certain level, although a gradual increase in the exposure to the traumatic events over time, irrespective of other important factors such as work experience. Based on the results in the study, no significant relationship was observed between work experience as a categorical variable and the total PCL-5 score, while work experience had a protective effect against PTSD in the multivariate linear regression after adjusting others variable (t= -3.23, p=0.001). The result is in line with that of other studies demonstrating the relationship between work experience and PTSD (27, 28).

Furthermore, a positive correlation was reported between the number of missions and the total PCL-5 score (β= 0.07) based on the multivariate linear regression after adjusting the variables. The result may reflect the effect of more exposure to traumatic events on PTSD. In another study in South Africa, the same relationship was observed between exposure to traumatic incidents and prevalence of mental health problems among emergency medical care personnel (29). In addition, the results are in line with some other studies in which it was reported that ongoing exposure and gaining enough experience simultaneously can increase the technician's ability to adapt, and develop resilience to stress over traumatic events (30, 31).

 The history of previous training and psychological debriefing sessions on managing and controlling stress in the prehospital emergency was regarded as another factor which was evaluated in the present study. Among the 259 participants, almost 54% had previous training. However, no significant relationship was reported between previous training and the total PCL-5 score (t= -0.83, P=0.319). The results of other studies indicated a considerable difference in the effect of training on reducing the stress among staff. For example, some studies emphasized that regular counseling or defusing sessions, as well as psychological debriefing and Critical Incident Stress Management (CISM), which is considered as an adaptive and short-term psychological helping-process, can prevent PTSD symptoms in personnel (32, 33). However, the results of a systematic review study showed that psychological debriefing has no preemptive effect on the PTSD incidence while Cognitive Behavior Therapy (CBT) for four weeks or more may prevent the development of trauma-related psychological disorders (34).

The level of education was another factor whose possible effect on the total PCL-5 score was evaluated (p=0.935) because the personnel's job in the EMS of Iran may not be related to the capability, skills, and training they have acquired. All-Advanced Life Support (ALS) and Basic Life Support (BLS) are performed by technicians with the same title (EMT), skill, and job responsibilities, which result in varying stress reactions. However, EMTs are divided into several levels in terms of training and clinical skills they have acquired such as EMT-B (Basic), EMT-I (Intermediate), and AEMT (Advanced) (35). For example, Minnie et al. reported that EMTs with a BLS and ILS qualification find all prehospital emergencies more traumatizing than those with an ALS qualification, and the difference observed was considerable for road traffic incidents (36). Therefore, in these countries, EMTs are dispatched to basic and advance emergencies in accordance with their skills and abilities, which makes the technicians more adaptable in the face of traumatic events, and accordingly they experience less stress than the other EMTs.

Finally, the results of the present study suggest that EMS authorities should be aware of some modifiable risk factors related to PTSD in order to adopt a proper follow-up and take preventive measures for EMTs at risk. 

Therefore, conducting a qualitative study to uncover potential stresses in exposure to the variety of prehospital emergency bases is essential. In addition, it is possible to reduce PTSD incidence in EMTs by changing some factors such as reducing the number of shifts, as well as increasing the staff’s experience by exposing them to traumatic events in a simulated environment.

**Table 1:** Relationship between the mean total PCL-5 score of each cluster with having or not having post-traumatic stress disorder (PTSD)

**PCL-5 Clusters**	**Total**	**PTSD**		P *
		NO (n = 207)	YES (n = 52)	
**Intrusion **	4.98 (3.08)	4.06 (2.48)	8.67 (2.40)	< 0.001
**Avoidance**	2.25 (1.70)	1.85 (1.42)	3.85 (3.62)	P<0.001
**Negative alterations in cognitions **	7.42 (4.63)	5.85 (3.40)	13.67 (3.47)	P<0.001
**Alterations in arousal and reactivity **	6.91 (4.15)	5.68 (3.44)	11.83 (2.92)	P<0.001

*: Mann-Whitney test. Data are presented as mean (Standard deviation).

**Table 2:** Relationship between the personal/work-related characteristics with the total PCL-5 score as well as the mean PCL-5 scores of each cluster

**Characteristics**	**n (%)**	**The Mean (SD) PCL-5 Cluster Scores**				**Total**	**P Value**
		**Intrusion**	**Avoidance**	**Cognition**	**Arousal**		
**Age (years)**							
≤ 30	92 (35.5)	6.61) 3.26)	2.88 (1.78)	8.92 (5.43)	8.61 (4.49)	27.02 (12.70)	< 0.001
31-40	134 (51.7)	3.73 (2.59)	1.77 (1.49)	6.02 (3.91)	5.50 (3.67)	17.08 (9.42)	
> 40	33 (12.7)	5.55 (2.03	2.45 (1.67)	8.91 (2.93)	7.91 (2.81)	24.82 (7.00)	
**Work experience (years)**							
≤ 10	166 (64.1)	5.47 (3.36)	2.42 (1.75)	7.90 (5.13)	7.49 (4.43)	23.31 (12.45)	0.204
11-20	89 (34.4)	4.20 (2.92)	2.02 (1.55)	6.69 (3.44)	5.98 (3.41)	18.91 (8.62)	
> 20	4 (1.5)	2.25 (0.5)	0.25 (0.5)	4.00 (2.44)	3.75 (2.06)	10.25 (4.39)	
**Marital status**							
Single	83 (32)	4.78 (3.03)	2.18 (1.75)	7.27 (4.52)	6.74 (4.21)	21.02 (11.73)	< 0.001
Maried	171 (66)	5.43 (3.23)	2.40 (1.61)	7.65 (4.89)	7.33 (4.12)	22.81 (11.09)	
Divorced	5 (1.9)	4.60 (1.14)	2.20 (1.70)	8.80 (4.63)	5.80 (4.15)	21.40 (6.34)	
**Employment status**							
Formal	97 (37.5)	4.43 (2.25)	2.11 (1.63)	7.09 (3.45)	6.49 (3.60)	20.15 (8.86)	0.282
Unformal	162 (62.5)	5.31 (3.45)	2.33 (1.74)	7.62 (5.21)	7.16 (4.43)	22.46 (12.70)	
**Degree**							
EMT	130 (50.2)	5.29 (3.50)	2.50 (1.88)	7.57 (5.03)	6.79 (4.30)	22.20 (12.51)	0.935
Nurse	61 (23.6)	5.11 (3.05)	2.09 (1.65)	7.57 (5.00)	6.86 (4.42)	21.63 (12.68)	
Operation	35 (13.5)	4.09 (2.66)	1.97 (1.48)	7.39 (4.01)	6.79 (3.75)	20.30 (9.91)	
Anastesia	33 (12.7)	4.74 (2.16)	1.97 (1.32)	7.03 (3.85)	7.26 (3.94)	21.00 (9.02)	
**Base location**							
Urban	165 (63.7)	5.17 (3.13)	2.35 (1.76)	7.59 (4.74)	7.23 (4.09)	22.38 (11.22)	0.146
Road	69 (26.6)	4.87 (3.00)	2.14 (1.61)	7.48 (4.75)	6.64 (4.49)	21.13 (12.42)	
Air	25 (9.7)	4.08 (3.05)	1.92 (1.52)	6.16 (3.42)	5.56 (3.40)	17.72 (9.54)	
**Number of shift (per month)**							
≤ 8	20 (7.7)	1.80 (1.60)	0.65 (0.98)	2.00 (0.00)	1.80 (0.61)	6.45 (2.11)	< 0.001
> 8	239 (92.3)	5.25 (3.03)	2.38 (1.68)	7.78 (4.51)	7.34 (4.03)	22.78 (10.99)	
**Number of missions (in the last month)**							
≤ 80	109 (39.4)	4.71 (3.00)	2.08 (1.52)	7.01 (4.23)	6.43 (4.16)	20.23 (11.34)	0.098
> 80	157 (60.6)	5.17 (3.13)	2.36 (1.80)	7.69 (4.87)	7.22 (4.12)	22.49 (11.47)	
**Previous training**							
Yes	139 (53.7)	4.76 (3.00)	2.39 (1.69)	7.37 (4.68)	6.53 (3.93)	21.01 (11.25)	0.375
No	120 (46.3)	5.25 (3.17)	2.09 (1.70)	7.48 (4.60)	7.35 (4.36)	22.24 (11.69)	

SD: standard deviation.

## Conclusions

The results indicated that the prevalence of PTSD among EMT personnel of Hamedan province is high. Negative alterations in cognition and avoidance were the most and least common clusters, respectively. EMTs with the age of ≤ 30 years, work experience of ≤ 10 years, married status, informal employment, emergency medical technician's degree, and more than 8 shifts per month, as well as no previous training history had a higher total PCL-5 score.

## Limitations

The results of the present study may not be generalizable to other contexts. In the present study individuals who had previously been diagnosed with PTSD or recovered were excluded, which could lead to an underestimation or overestimation of PTSD rate in the present study. Also, the findings may be gender biased due because of the lack of female technicians in the pre-hospital system considered as sample in the present study.

No study has focused on determining the proper cut-off point for the PCL-5 instrument in the EMS staff in an Iranian context. Hence, the cut-off point used to determine PTSD in the present study may not give an accurate prediction of the participants at risk. Variation in estimating the levels of PTSD can be related to the use of different PTSD assessment tools in the prehospital emergency (22). Few studies have been conducted using the PCL-5 tool for examining and detecting PTSD among EMTs. Therefore, comparing the results of the present study with other studies may produce some type of bias (39).

## References

[1] Kilic C, Inci F (2015). [Traumatic Stress in Emergency Medical Technicians: Protective Role of Age and Education]. Turk psikiyatri dergisi = Turkish journal of psychiatry..

[2] Khashaba EO, El-Sherif MAF, Ibrahim AA-W, Neatmatallah MA (2014). Work-Related Psychosocial Hazards Among Emergency Medical Responders (EMRs) in Mansoura City. Indian Journal of Community Medicine : Official Publication of Indian Association of Preventive & Social Medicine..

[3] Geronazzo-Alman L, Eisenberg R, Shen S, Duarte CS, Musa GJ, Wicks J (2017). Cumulative exposure to work-related traumatic events and current post-traumatic stress disorder in New York City's first responders. Comprehensive psychiatry..

[4] Ahmadizadeh MJ, Ahmadi K, Eskandari H, Falsafinejad MR, Borjali A, Anisi J (2010). Improvement in quality of life after exposure therapy, problem solving and combined therapy in chronic war-related post traumatic stress disorder: Exposure therapy, problem solving and combined therapy in war-related PTSD. Procedia - Social and Behavioral Sciences..

[5] Conrad D, Wilker S, Pfeiffer A, Lingenfelder B, Ebalu T, Lanzinger H (2017). Does trauma event type matter in the assessment of traumatic load?. European Journal of Psychotraumatology..

[6] Petrie K, Milligan-Saville J, Gayed A, Deady M, Phelps A, Dell L (2018). Prevalence of PTSD and common mental disorders amongst ambulance personnel: a systematic review and meta-analysis.

[7] Lima Ede P, Assuncao AA (2011). [Prevalence and factors associated with Posttraumatic Stress Disorder (PTSD) in emergency workers: a systematic literature review]. Revista brasileira de epidemiologia = Brazilian journal of epidemiology..

[8] Berger W, Coutinho ES, Figueira I, Marques-Portella C, Luz MP, Neylan TC (2012). Rescuers at risk: a systematic review and meta-regression analysis of the worldwide current prevalence and correlates of PTSD in rescue workers. Social psychiatry and psychiatric epidemiology..

[9] Iranmanesh S, Tirgari B, Bardsiri HS (2013). Post-traumatic stress disorder among paramedic and hospital emergency personnel in south-east Iran. World Journal of Emergency Medicine..

[10] Weathers FW LB, Keane TM, Palmieri PA, Marx BP, Schnurr PP (2010 at www). The PTSD Checklist for DSM-5 (PCL-5) Scale available from the National Center for PTSD.

[11] Statistical Manual of Mental Disorders-Fifth Edition (PCL-5) in veterans": Correction to Bovin et al, Psychometric properties of the PTSD Checklist for Diagnostic (2017). Psychological assessment..

[12] Sveen J, Bondjers K, Willebrand M (2016). Psychometric properties of the PTSD Checklist for DSM-5: a pilot study. European Journal of Psychotraumatology..

[13] Ashbaugh AR, Houle-Johnson S, Herbert C, El-Hage W, Brunet A (2016). Psychometric Validation of the English and French Versions of the Posttraumatic Stress Disorder Checklist for DSM-5 (PCL-5). PLoS One..

[14] Sadeghi M, Taghva A, Goudarzi N, Rah Nejat AM (2016). Validity and Reliability of Persian Version of “Post-Traumatic Stress Disorder Scale” in War Veterans. Iranian Journal of War and Public Health..

[15] Verhey R, Chibanda D, Gibson L, Brakarsh J, Seedat S (2018). Validation of the posttraumatic stress disorder checklist – 5 (PCL-5) in a primary care population with high HIV prevalence in Zimbabwe. BMC Psychiatry..

[16] Mills LD, Mills TJ (2005). Symptoms of post-traumatic stress disorder among emergency medicine residents. J Emerg Med..

[17] van der Ploeg E, Kleber RJ (2003). Acute and chronic job stressors among ambulance personnel: predictors of health symptoms. Occupational and environmental medicine..

[18] Kerai SM, Khan UR, Islam M, Asad N, Razzak J, Pasha O (2017). Post-traumatic stress disorder and its predictors in emergency medical service personnel: a cross-sectional study from Karachi, Pakistan. BMC emergency medicine..

[19] Adams RE, Boscarino JA (2006). Predictors of PTSD and Delayed PTSD After Disaster: The Impact of Exposure and Psychosocial Resources. The Journal of nervous and mental disease..

[20] Rahmani A, khodaei R, Mahmodkhani S, Moslemi M, Gharagozlou F, Ahmadnezhad I (2013). Investigation of Occupational Stress and its Relationship with the Demographic Characteristics of Workers in Ilam, Iran. Electronic physician..

[21] Skogstad M, Skorstad M, Lie A, Conradi HS, Heir T, Weisaeth L (2013). Work-related post-traumatic stress disorder. Occupational medicine (Oxford, England)..

[22] Petrie K, Milligan-Saville J, Gayed A, Deady M, Phelps A, Dell L (2018). Prevalence of PTSD and common mental disorders amongst ambulance personnel: a systematic review and meta-analysis.

[23] Fjeldheim CB, Nothling J, Pretorius K, Basson M, Ganasen K, Heneke R (2014). Trauma exposure, posttraumatic stress disorder and the effect of explanatory variables in paramedic trainees. BMC emergency medicine..

[24] Schiszler B, Karamanne Pakai A, Szabo Z, Raposa LB, Ponusz R, Radnai B (2016). [Examination of work-related stress and coping strategies among ambulance- and air-ambulance workers]. Orvosi hetilap..

[25] Short NA, Allan NP, Schmidt NB (2017). Sleep disturbance as a predictor of affective functioning and symptom severity among individuals with PTSD: An ecological momentary assessment study. Behaviour research and therapy..

[26] Jehan S, Zizi F, Pandi-Perumal SR, Myers AK, Auguste E, Jean-Louis G (2017). Shift Work and Sleep: Medical Implications and Management. Sleep medicine and disorders : international journal..

[27] Onyedire NG, Ekoh AT, Chukwuorji JC, Ifeagwazi CM (2017). Posttraumatic stress disorder (PTSD) symptoms among firefighters: Roles of resilience and locus of control. Journal of Workplace Behavioral Health..

[28] Khan K, Charters J, Graham TL, Nasriani HR, Ndlovu S, Mai J (2017). A Case Study of the Effects of Posttraumatic Stress Disorder on Operational Fire Service Personnel Within the Lancashire Fire and Rescue Service. Safety and Health at Work.

[29] Ward CL, Lombard CJ, Gwebushe N (2006). Critical incident exposure in South African emergency services personnel: prevalence and associated mental health issues. Emergency medicine journal : EMJ..

[30] Lee J-K, Choi H-G, Kim J-Y, Nam J, Kang H-T, Koh S-B (2016). Self-resilience as a protective factor against development of post-traumatic stress disorder symptoms in police officers. Annals of Occupational and Environmental Medicine..

[31] Kang X, Fang Y, Li S, Liu Y, Zhao D, Feng X (2018). The Benefits of Indirect Exposure to Trauma: The Relationships among Vicarious Posttraumatic Growth, Social Support, and Resilience in Ambulance Personnel in China. Psychiatry Investigation..

[32] Bledsoe BE (2003). Critical incident stress management (CISM): benefit or risk for emergency services?. Prehospital emergency care : official journal of the National Association of EMS Physicians and the National Association of State EMS Directors..

[33] Lim JJ, Childs J, Gonsalves K (2000). Critical incident stress management. AAOHN journal : official journal of the American Association of Occupational Health Nurses..

[34] Kornor H, Winje D, Ekeberg O, Johansen K, Weisaeth L, Ormstad SS NIPH Systematic Reviews: Executive Summaries. Psychosocial Interventions After Crises and Accidents. Oslo, Norway: Knowledge Centre for the Health Services at The Norwegian Institute of Public Health.

[35] Jacobs PE, Grabinsky A (2014). Advances in prehospital airway management. International Journal of Critical Illness and Injury Science..

[36] Minnie L, Goodman S, Wallis L (2015). Exposure to daily trauma: The experiences and coping mechanism of Emergency Medical Personnel. A cross-sectional study. African Journal of Emergency Medicine..

[37] Knaak S, Mantler E, Szeto A (2017). Mental illness-related stigma in healthcare: Barriers to access and care and evidence-based solutions. Healthcare Management Forum..

[38] Alexander DA, Klein S (2001). Ambulance personnel and critical incidents: impact of accident and emergency work on mental health and emotional well-being. The British journal of psychiatry : the journal of mental science..

[39] McGauran N, Wieseler B, Kreis J, Schüler Y-B, Kölsch H, Kaiser T (2010). Reporting bias in medical research - a narrative review. Trials..

